# Machine‐Learning Applications in Frontotemporal Dementia: Challenges, Prospects and Viable Clinical Applications

**DOI:** 10.1111/ene.70319

**Published:** 2025-09-09

**Authors:** Peter Bede, We Fong Siah, Jan Kassubek

**Affiliations:** ^1^ Computational Neuroimaging Group (CNG), School of Medicine Trinity College Dublin Dublin Ireland; ^2^ Department of Neurology St James's Hospital Dublin Ireland; ^3^ Department of Neurology University of Ulm Ulm Germany

**Keywords:** artificial intelligence, big data, biomarkers, frontotemporal dementia, machine‐learning, magnetic resonance imaging, neurodegeneration, neuroimaging

Machine‐learning (ML) models and ‘big data’ have revolutionised data interpretation in neurodegenerative conditions. We are witnessing a paradigm shift from large descriptive studies describing group‐level phenomena, genotype‐associated traits and phenotype‐specific biomarker signatures to studies meaningfully interpreting data from individual patients and accurately classifying them into relevant diagnostic, phenotypic and prognostic categories. In the era of biomarker‐supported pharmaceutical trials, precision clinical monitoring and validated prognostic indicators, ML models are likely to play a central role in clinical decision making and monitoring for years to come.

Low‐incidence neurodegenerative disorders, such as frontotemporal dementia (FTD), are notoriously difficult to diagnose early, soon after symptom onset, and the emphasis initially is on ruling out alternative structural, neuroinflammatory, paraneoplastic or more common neurodegenerative conditions that can mimic FTD. FTD and ALS have overlapping clinical, radiological, molecular and genetic characteristics, and comorbid ALS‐FTD is a well‐recognised entity [1]. FTD spectrum disorders manifest insidiously after a long presymptomatic phase with subtle, but relentlessly progressive symptoms, and develop into distinctive FTD phenotypes with unique neuroimaging signatures [2]. Diagnostic delay in FTD has considerable practical implications, such as the delayed introduction of supportive measures, delayed genetic screening and genetic counselling of family members, and ultimately, protracted recruitment into pharmaceutical trials. With practical clinical objectives in mind, a multitude ML‐based applications has been proposed recently to distinguish FTD from AD radiologically and differentiate various FTD subtypes [3].

The clinical utility of a variety of supervised and unsupervised statistical models has been explored in recent studies. Based on a regression approach, a recent study demonstrated that the combination of morphological and clinical metrics facilitates disease phenotyping in FTD [4]. Advanced ML models have successfully interrogated large longitudinal datasets to delineate spatiotemporal neuroanatomical phenotypes within the primary progressive aphasia spectrum [5]. Other groups have evaluated quantitative MRI parameters and fluid biomarkers to assess the interaction between regional atrophy patterns and ‘wet’ biomarker profiles in various FTD phenotypes [6].

In this edition of the *European Journal of Neurology*, Spinelli et al. [7] present a thought‐provoking classification study in FTD. They not only describe unique structural trajectories associated with specific FTD phenotypes, but they also implement a multi‐class support vector machine (SVM) model to categorise individual patients with good accuracy into disease phenotypes using both baseline and follow‐up data. Their study also demonstrated the potential of quantitative MRI to consolidate the distinct profile of low‐incidence clinicopathological entities such as semantic behavioural variant FTD (sbvFTD). Their study exemplifies recent trends in using ML for clinically meaningful data interpretation. SVM has also been implemented in another recent FTD study for the analysis of structural and diffusion metrics in PPA, supporting the broader application of ML models for pattern recognition and longitudinal tracking of disease progression in vivo [8].

The unique anatomical patterns of radiological involvement with the predilection to specific brain regions and the relative sparing of others offer unprecedented opportunities for automated case classification into relevant phenotypic categories. As a departure from small, single‐centre studies using binary classification models, more recent ML studies of FTD spectrum disorders use complex unsupervised techniques and not only allocate individuals into relevant diagnostic categories, but reflect on prognosis and predict phenoconversion in asymptomatic carriers of specific genetic variants [9]. Beyond potential clinical applications, many of these studies confirm phenotype‐specific imaging signatures [10], underlying disease burden patterns and offer additional academic insights such as putative propagation mechanisms [8]. One of the prerequisites of successful ML studies is the curation of large, ‘well‐labelled’, training data sets. In recognition of the importance of high‐quality data sets, large local, national and international data repositories have been established in both FTD and ALS such as the GENFI, ALLFTD, NISALS, CALSNIC, Precision ALS, NEALS, etc. These repositories are invaluable resources for model training, testing, optimisation and validation. These initiatives have helped to overcome the sample size limitations of single‐centre studies and facilitated the evaluation of large, well‐characterised clinical cohorts. Some imaging consortia operate with strict data harmonisation procedures, whereas others serve primarily as a knowledge exchange forum to foster collaborations and multicentre research.

Machine‐learning represents an exciting and clinically useful frontier of FTD research, and the lessons of recent academic studies are likely to filter down to pragmatic everyday applications aiding clinical decision‐making and curtailing the notoriously long diagnostic journey that patients with neurodegenerative conditions currently face.

## Author Contributions

**Peter Bede:** conceptualization, writing – original draft. **We Fong Siah:** conceptualization, writing – original draft. **Jan Kassubek:** conceptualization, writing – original draft.

## Conflicts of Interest

The authors declare no conflicts of interest.

## Linked Articles

Distinctive progression patterns of brain structural damage aid classification of frontotemporal dementia variants, https://doi.org/10.1111/ene.70275.

## Data Availability

Data sharing is not applicable to this article as no datasets were generated or analysed during the current study.
